# Improving medicines management for people with dementia in primary care: a qualitative study of healthcare professionals to develop a theory-informed intervention

**DOI:** 10.1186/s12913-020-4971-7

**Published:** 2020-02-14

**Authors:** Heather E. Barry, Laura E. Bedford, Máiréad McGrattan, Cristín Ryan, A. Peter Passmore, A. Louise Robinson, Gerard J. Molloy, Carmel M. Darcy, Hilary Buchanan, Carmel M. Hughes

**Affiliations:** 1 School of Pharmacy, Queen’s University Belfast, Belfast, Northern Ireland, UK; 2 The School of Pharmacy and Pharmaceutical Sciences, Trinity College Dublin, Dublin, Ireland; 3 Centre for Public Health, Queen’s University Belfast, Belfast, Northern Ireland, UK; 4 Belfast Health & Social Care Trust, Belfast City Hospital, Belfast, Northern Ireland, UK; 5 Institute for Ageing and Institute for Health & Society, Newcastle University, Newcastle upon Tyne, UK; 6 School of Psychology, National University of Ireland, Galway, Ireland; 7 Western Health & Social Care Trust, Londonderry, UK; 8 Belfast, UK

**Keywords:** APEASE, Behaviour change, Dementia, Intervention, Medicines management, Primary care, Qualitative, Theoretical domains framework

## Abstract

**Background:**

People with dementia (PwD) face unique challenges with medicines management, yet little is known about these challenges from the perspectives of primary healthcare professionals, particularly general practitioners (GPs) and community pharmacists. Few medicines management interventions have been developed which are aimed at community-dwelling PwD. This study sought to develop an intervention to improve medicines management for PwD in primary care using a theory-informed approach.

**Methods:**

Semi-structured interviews were conducted with GPs (*n* = 15) and community pharmacists (*n* = 15) to explore participants’ views and experiences of medicines management for PwD, and their perceptions of barriers and facilitators to successful medicines management for PwD. The 14-domain Theoretical Domains Framework was the underpinning theoretical guide, allowing key theoretical domains to be identified and mapped to behaviour change techniques (BCTs) which are considered the ‘active ingredients’ of an intervention. Draft interventions were developed to operationalise selected BCTs and were presented to GPs and community pharmacists during task groups. Final selection of an intervention for feasibility testing was guided by feedback provided during these task groups and through application of the APEASE (Affordability, Practicability, Effectiveness/cost-effectiveness, Acceptability, Side-effects/safety, Equity) criteria.

**Results:**

Participants expressed a number of concerns about medicines management for PwD, particularly monitoring adherence to medication regimens and conducting medication review. Two draft interventions comprising selected BCTs (‘Modelling or demonstration of behaviour’; ‘Salience of consequences’; ‘Health consequences’; ‘Social and environmental consequences’; ‘Action planning’; Social support or encouragement’, ‘Self-monitoring of behaviour’) were developed, each targeting GPs and community pharmacists. Following the task groups and discussions within the research team, the community pharmacy-based intervention was selected for future feasibility testing. The intervention will target community pharmacists to conduct a medication review (incorporating an adherence check) with a PwD, delivered as an online video demonstrating key behaviours. The video will include feedback emphasising positive outcomes of performing the behaviours. Action planning and a quick reference guide will be used as complementary intervention components.

**Conclusions:**

A community pharmacist-based intervention has been developed targeting medicines management for PwD in primary care using a systematic, theory-informed approach. Future work will determine the usability and acceptability of implementing this intervention in clinical practice.

## Background

Medicines management is a broad concept, defined as encompassing ‘the entire way medicines are selected, procured, delivered, prescribed, administered and reviewed to optimise the contribution that medicines make to producing informed and desired outcomes of patient care’ [1]. In this study, we considered the following as essential components of medicines management: prescribing, dispensing, administration, adherence, and medication review. People with dementia (PwD) face unique challenges with medicines management which may increase their risk of negative outcomes, such as adverse drug events, hospitalisation and mortality. Multimorbidity is highly prevalent in PwD [2–4]; consequently, PwD may be subject to complex medication regimens, polypharmacy and potentially inappropriate prescribing [5–7]. PwD may find medication regimens difficult to manage, and due to impairments in cognition and communication, adherence to medication may be poor [8, 9]. Management of behavioural and psychological symptoms of dementia (BPSD) may also present challenges to healthcare professionals (HCPs) [10, 11] and those administering medications [9, 12]. Most PwD (61%) in the United Kingdom (UK) live in the community [13], and are managed within primary care. PwD are reported to have high health service usage in terms of primary care consultations and prescribing [14], and many PwD living at home receive assistance with their medicines from formal and/or informal carers [15, 16].

At the time of study planning, there had been limited work in this area, with research focusing predominantly on antipsychotic drug use in PwD particularly care home residents, and medicines use in advanced dementia. A systematic review of the effectiveness of medicines management interventions for PwD in primary care [17], highlighted the small number of studies (*n* = 3), concluding that future interventions must target community-dwelling PwD and take a holistic and multidisciplinary approach to medicines management.

Intervention development has been criticised within the literature, due to lack of clarity on the process undertaken [18], making subsequent evaluation difficult [19]. The Medical Research Council (MRC) guidance for complex interventions [20] provides a robust and systematic approach, which had been used by individual team members previously [21–23]. It emphasises identifying existing evidence in the area, and developing a theoretical understanding of the likely process of change to inform intervention design [22]. Our systematic review [17] highlighted a lack of theory-based interventions. We had already undertaken a pharmacoepidemiological study of prescribing appropriateness in community-dwelling PwD in Northern Ireland (NI) to extend the evidence base. This demonstrated a high prevalence of polypharmacy among PwD, and revealed common instances of potentially inappropriate prescribing [7]. The current study aimed to take a theoretical approach to develop an intervention to improve medicines management for PwD in primary care, engaging key stakeholders (PwD, their carers, general practitioners (GPs), and community pharmacists) in the process. This paper focuses on HCP stakeholders (i.e. GPs and community pharmacists); findings from qualitative work with PwD and their carers will be reported separately.

The Theoretical Domains Framework (TDF) provided the underpinning theoretical guide for the study [24]. It comprises 14 domains of theoretical constructs related to behaviour change (Additional file 1), which may act as facilitators or barriers to an individual’s behaviour. The TDF was used to understand what needed to change in order to achieve successful medicines management for PwD [25]. Key theoretical domains, considered the ‘mechanisms of change’, were mapped to Behaviour Change Techniques (BCTs), the ‘active components’ of an intervention – if enacted appropriately, the selected BCTs have potential to bring about the desired behaviour change [26]. Intervention drafting, and subsequent selection of final intervention components, was informed by the context and any other pertinent constraints (e.g. timescale, budget) regarding the application and mode of delivery of BCTs in the given setting [27].

The objectives of the study were therefore to: (1) identify barriers and facilitators of successful medicines management from the perspectives of GPs and community pharmacists; (2) identify behaviours and key theoretical domains to target to achieve the desired changes; (3) map these key domains to corresponding BCTs; and (4) develop an intervention to improve medicines management for PwD in primary care, incorporating previously selected BCTs.

## Methods

### Design and setting

Face-to-face, semi-structured interviews were conducted with GPs and community pharmacists across NI. Ethical approval was obtained from East of England – Cambridgeshire and Hertfordshire Research Ethics Committee (15/EE/0103). The study is reported according to the Consolidated criteria for reporting qualitative research (COREQ) checklist [28].

### Participant sampling and recruitment

We drew on previous experience of conducting research with primary HCPs [22]. The Primary Care sub-group of the NI Clinical Research Network (NICRN) assisted with recruitment. General practices were purposively sampled and recruited from a range of geographical locations across NI. A computer-generated random sample of practices from each of five Health and Social Care (HSC) Trusts (main administrative health areas in NI) was contacted by telephone by a NICRN research nurse. We recruited two practices (one urban, one rural) per HSC Trust, and GPs from each practice were invited to take part in an interview (with the aim of interviewing at least one GP per practice). Recruited practices were then asked to identify community pharmacies which dispensed most of the prescriptions they issued, and pharmacists from these pharmacies were also invited to participate in the study (again, with the aim of interviewing at least one pharmacist per community pharmacy). There were no specific inclusion or exclusion criteria for recruitment of HCPs.

### Data collection

Interviews were conducted by the researchers (HB, MM; both qualified pharmacists) at the participant’s place of work (i.e. GP surgery or community pharmacy). All participants provided written informed consent. Interview topic guides were based on the 14 domains of the TDF [24] and developed following research team discussion. Whilst a separate topic guide was developed and piloted for each HCP group (Additional files 2 and 3), both followed a similar format covering three main areas. Participants were provided with an explanation of the term medicines management, and asked to reflect upon their own experiences and what they felt their roles/responsibilities were in relation to medicines management for PwD. Participants were then asked focused questions (with prompts when appropriate) guided by the 14 TDF domains to obtain their perceptions of the barriers and facilitators to achieving successful medicines management for PwD. Lastly, participants were asked their views about potential intervention components and outcome measures for inclusion in future intervention studies. All participants were offered an honorarium of £50 and awarded a certificate of participation.

### Data analysis

Interviews were audio recorded, transcribed verbatim, and anonymised. Codes were assigned to differentiate between general practitioner (GP) or community pharmacist (CP) participants, together with a two-digit identification number. Data were managed using NVivo 11 software [29].

Each transcript was analysed independently by two researchers (HB, MM). Data analysis comprised a number of stages, modelled on approaches used previously [21–23]. The primary focus of analysis was the TDF-related data. The framework method [30] was used to deductively code and organise data into categories that mirrored the 14 TDF domains [24]. The researchers met face-to-face to compare and agree coding; discrepancies were resolved through discussion with a third analyst (CH). Summarised data were charted to generate a framework matrix using a Microsoft Excel spreadsheet [30], which included illustrative quotes. Content analysis [31] of this matrix identified barriers and facilitators perceived to influence the achievement of successful medicines management for PwD within each TDF domain. Due to complex interaction between the different behaviours involved in the medicines management process, we spent time focusing on each of the ‘target behaviours’ [27] identified by HCPs during the interviews. These were specified in the form of ‘narratives’ concentrating on answering the following questions [27]: *Who* needs to perform the behaviour? *What* does the person need to do differently to achieve the desired change? *When, where, how often* and *with whom* will they do it? A summary of findings was produced for each HCP participant group outlining the barriers, facilitators and problems/priorities, linked to the target behaviours discussed under each theoretical domain. These summaries were reviewed and discussed by members of the research team.

#### Identification of key theoretical domains

We sought to identify key theoretical domains for each target behaviour through discussion and consensus. This approach was guided by previous research [21, 22], whereby the extent to which sections of interview transcripts were coded to each domain was viewed as a crude indicator of relevance; summary documents were then used to determine whether participants related the domain to the target behaviour [32]. Consideration was also given to barriers and facilitators within relevant domains that could feasibly be targeted as part of a future intervention based on available project resources.

#### Triangulation

Data source triangulation [33] was conducted, using evidence gathered from the different participant groups during the course of the study. We compared and contrasted participants’ perceptions of barriers and facilitators within each of the theoretical domains, which helped to inform decision-making as to how BCTs could be operationalised as part of a future intervention.

#### Mapping of key theoretical domains to BCTs

The process used to map key theoretical domains to BCTs was informed by methods used previously [22, 23], utilising established BCT mapping taxonomies [34, 35]. The matrix published by Cane et al. [34] was used initially, however we also referred to the matrix by Michie et al. [35] because in some instances, no BCTs could be clearly linked to domains within the Cane et al. matrix, e.g. ‘Social/professional role and identity’. The BCT mapping and selection process was informed by discussion within the research team, guided by the interview data, to reach a consensus-based decision. Other factors considered during the selection process included the applicability of the BCT to the target population, the feasibility of operationalising the BCT in a future intervention delivered within primary care and within the scope of the project.

#### Draft intervention development

Following identification of BCTs, consideration was given as to how they could be applied in practice. In accordance with previously published guidance, mode of delivery and intervention content were considered [25]. From the outset of study planning, it was anticipated that two draft interventions (one delivered by GPs and the other by community pharmacists) would be developed. Both interventions were informed by the interview data, local context, preceding research [7], the multidisciplinary research team’s professional expertise, as well as our experience of operationalising BCTs in previous studies [21–23, 36].

#### Task group work and selection of final intervention components

Task groups were conducted with GPs and community pharmacists to obtain their views on draft intervention outlines and to aid selection of the final intervention for future feasibility testing. Task groups are a hybrid focus group intended to generate both ‘conventional’ qualitative data and sets of principles or proposals for action grounded in the experience of group members [37, 38]. Those GPs and pharmacists who had been recruited previously for interviews were approached and invited to contribute. Task group content was developed based on previous studies that have used this approach [37, 38]. To initiate open discussion and establish a degree of consensus regarding key issues, participants were presented with interview statements and asked to categorise these as ‘true’, ‘false’ or ‘interesting’. Participants reviewed and commented on the aforementioned ‘narratives’ of identified target behaviours. Finally, participants appraised draft intervention outlines using the APEASE criteria (Affordability, Practicability, Effectiveness and cost effectiveness, Acceptability, Side-effects/safety, Equity), which were developed to guide context-based decisions on intervention content and delivery [27]. Task group discussions were audio-recorded and analysed using thematic analysis to identify themes and subthemes in relation to proposed intervention components. Three members of the research team (HB, LB, CH) met to discuss and agree on final intervention components, with consideration given to feasibility of implementation within the scope of the project (e.g. time and resource restrictions).

## Results

### Sample characteristics

Fifty-two general practices and 18 community pharmacies were contacted about the study. Thirty participants (*n* = 15 GPs, *n* = 15 community pharmacists) were recruited from nine general practices and 15 community pharmacies across NI between October 2015 and March 2016. Whilst 10 general practices were recruited initially, GPs from one practice later refused to take part in an interview due to time constraints. Demographic characteristics of HCPs are shown in Table 1. Interviews lasted between 35–60 min (GPs) and 33–80 min (community pharmacists).

**Table 1:** Healthcare professional participant characteristics

	General practitioners (*n* = 15)	Community pharmacists (*n* = 15)
Participant gender		
Male	7	8
Female	8	7
Years of professional practice (range)	5–30	1–27
HSC Trust area		
Belfast	3	4
Northern	4	3
Southern	5	2
South Eastern	2	3
Western	1	3

### Summary of findings from TDF analysis

GPs discussed medicines management for PwD in terms of two main responsibilities (i.e. target behaviours) they felt they had: prescribing and conducting medication review (‘Social/professional role and identity’). Community pharmacists, however, predominantly discussed conducting medication review and monitoring adherence in these patients (‘Social/professional role and identity’). Therefore, provided below is a summary of the factors within each of the theoretical domains that were perceived to influence each of these medicines management behaviours (i.e. prescribing, conducting medication review, monitoring adherence).

Both HCP groups acknowledged the benefits of optimising medicines management for PwD (‘Beliefs about consequences’). There was concern amongst GP participants about polypharmacy. However, there was recognition of the benefits of deprescribing (the process of tapering, withdrawing, discontinuing or stopping medicines) as part of a medication review:

“ *… often I feel that patients might benefit from coming off tablets than being on a lot of things, maybe that would be something in the future that happens more?*” [GP_15]

Both GPs and community pharmacists believed that adherence was poor amongst PwD, with particular concern about over-adherence (‘Beliefs about consequences’):

“*I think you just have to assume it’s* [adherence] *not going to be very good. They’re always at risk aren’t they? Even if it’s* [medication] *in a weekly dispensing pack, there’s many ones that open up the wrong day and take two lots* [of tablets]*.*” [GP_13]

“ *… because you don’t know if they’re going to not use, or if they’re going to overuse … My concern would also be the overdosing on medicines as well*.” [CP_13]

Clinical knowledge was discussed as a facilitator by both HCP groups when contributing to medicines management for PwD (‘Knowledge’). In particular, pharmacists felt their knowledge was hindered by lack of access to full medication histories (‘Environmental context and resources’) affecting their ability to conduct comprehensive medication review (‘Beliefs about capabilities’). Both GPs and pharmacists talked about the importance of having knowledge of patients’ personal and social circumstances in order to understand the mechanisms of support available to patients with their medicines (‘Knowledge’, ‘Social influences’):

“*Whenever you prescribe for an individual, you’re looking at the whole situation*.” [GP_01]

“*It’s very good to understand their family situation and who is looking in on them … just checking the patient isn’t becoming isolated and that there are people out there who can support them*.” [CP_03]

This was facilitated by building good relationships with patients and carers (‘Skills’) and, for community pharmacists in particular, a break in continuity of pharmacist care was a barrier to this (‘Social/professional role and identity’):

“ *… you really need to know the patient. There’s no point in one pharmacist dealing with the patient one week and another pharmacist dealing with them the next week*” [CP_11]

A small number of pharmacists discussed difficulties in dealing with challenging behaviours that may be exhibited by PwD (e.g. agitation, aggression) and the lack of training in this area (‘Skills’).

Both HCP groups felt that carers significantly influenced their clinical behaviours and this impacted a number of theoretical domains. Carers were considered a reliable resource (‘Social influences’) credited with bringing HCPs’ attention to medicines-related issues (‘Memory, attention and decision processes’), and a vital part of strategies used to improve medicines management for PwD (‘Behavioural regulation’). Participants described having more confidence when addressing medicines management issues with patients if a carer was present (‘Beliefs about capabilities’):

“*Carers often feedback to us if they’re* [patient] *not taking it* [medication] *correctly, in which case we try and address it.”* [GP_05]

“*If you’ve done something and want to follow up on it, you can speak to a carer or somebody that you can rely on for them to phone you back, you need to put some sort of safety net there.*” [GP_15]

“*…family members know the patient better than anyone, so they can advise you what is going to suit a particular patient better.*” [CP_09]

Both HCP groups felt that optimising medicines for PwD was part of their professional responsibility (‘Social/professional role and identity’). Community pharmacists felt that their accessibility within primary care was a facilitator:

“*…we may deal with these patients more than any other healthcare professional. They might not see their GP as often.*” [CP_02]

Whilst each HCP group acknowledged the good working relationship they had with the other HCP group as a facilitator to achieving optimal patient care, some professional boundaries were discussed (‘Social/professional role and identity’). GPs focused on the boundaries they encountered with secondary care, and how this influenced their professional confidence when monitoring prescribing of dementia drugs (‘Beliefs about capabilities’):

“*There’s a bit of a cut-off between GPs and consultants…I don’t feel there’s a very natural relationship*.” [GP_07]

“*…because specialist dementia drugs are secondary care initiated, I am a little bit… more hesitant, because how do I measure whether they’re working or not?*” [GP_10]

Some community pharmacists mentioned professional boundaries with GPs. However, conversely, GPs were positive about community pharmacists’ input with these patients (‘Social influences’/‘Social/professional role and identity’), with many reporting that community pharmacists were a useful resource (‘Environmental context and resources’) often bringing medicines management-related issues to their attention (‘Memory, attention and decision processes’). GPs also recognised the role of practice-based pharmacists in the future, particularly with regard to prescribing and medication review (‘Environmental context and resources’):

“*…it’s not the first time I’ve prescribed something and the chemist says, ‘Are you sure you want to prescribe this?”* [GP_13]

“*There is certainly a role which needs to be developed for a pharmacist or prescribing pharmacist in surgeries to review all* [dementia] *patients, but particularly* [those] *on numerous drugs, say five, ten or more items*” [GP_01]

A number of emotions were expressed by participants when discussing medicines management for PwD. Both HCP groups demonstrated empathy towards patients, but expressed concern about their vulnerability, describing feelings of anxiety and worry (‘Emotion’). Such feelings were heightened when dealing with patients alone, without a carer/family member present:

“*You do worry more with patients with dementia. You know, just, is it safe? It’s simple as that, is a medication safe, whatever they’re on.*” [GP_15]

“*There are times when I am nervous. If it is the patient themselves, sometimes you just don’t know that what you’re saying is going in…*” [CP_09]

Some community pharmacists described feeling loss of control once PwD had left the pharmacy and were managing their medicines at home (‘Emotion’) which also influenced their professional confidence (‘Beliefs about capabilities’):

“*What the total unknown is when you give out medication is what really is happening…”* [CP_02]

“*We can be sure that we have given them the right medications with the right instructions and the right information, but after that it is beyond our control*” [CP_05]

Both HCP groups discussed the routine procedures embedded within their practices and pharmacies to monitor and review medicines management for PwD. These included the use of computer system prompts, notes, memos, and weekly dispensing/compliance aids (‘Memory, attention and decision processes’, ‘Behavioural regulation’). However participants also identified a number of barriers to optimising medicines management for PwD. Lack of time was one of the most frequently cited, particularly with regard to medication review, and was linked to the increasing complexity of patients’ needs and resultant heavy workload (‘Environmental context and resources’):

“*Primary care has changed whereby the patients that we are seeing tend to be complex, they tend to be elderly… to try and sort these patients in ten minutes is now becoming impossible*.” [GP_12]

“*We have a great desire… great intention…, but we just haven’t found ourselves with an awful lot of time to do them* [medication reviews].” [CP_05]

Some participants did not view PwD as any greater a priority than other patient groups (‘Goals’) and lack of financial reward or other incentives (‘Reinforcement’) was also cited as barrier:

*“My concern would be that those are not the only patients that are looked at…we have a much bigger problem going on in our practice. It is not just dementia.”* [GP_08]

“*Weekly dispensing isn’t money for the pharmacy anymore. It’s done at a cost to us.*” [CP_01]

### Identification of key theoretical domains

The narratives produced for each identified target behaviour (GPs: prescribing and conducting medication review; community pharmacists: conducting medication review and monitoring adherence) are provided in Additional file 4. The key theoretical domains identified against each target behaviour are shown in Table 2. Overall, twelve of the 14 domains were considered relevant to achieving appropriate medicines management for PwD – the domains ‘Optimism’ and ‘Intentions’ were not considered to be important because explicit links could not be made between beliefs expressed by HCPs and their clinical behaviour.

**Table 2:** Key theoretical domains identified by medicines management target behaviour for each healthcare professional (HCP) group

Theoretical domain	GP		Community pharmacist	
	Prescribing	Conducting medication review	Monitoring adherence	Conducting medication review
Knowledge	✓		✓	
Skills		✓	✓	✓
Memory, attention and decision processes		✓	✓	
Behavioural regulation	✓	✓	✓	
Social/professional role and identity				✓
Beliefs about capabilities	✓		✓	
Beliefs about consequences	✓		✓	✓
Goals	✓	✓	✓	
Reinforcement	✓	✓	✓	
Emotion	✓		✓	
Environmental context and resources	✓	✓	✓	✓
Social influences	✓	✓	✓	✓

### Mapping of theoretical domains to BCTs

There were 107 BCTs identified from the BCT mapping reference sources [34, 35]. Further detail on the mapping process and selection of BCTs is provided in Additional file 5. Seven BCTs were subsequently selected by the research team for inclusion in a future intervention involving GPs and/or community pharmacists to improve medicines management for PwD in primary care. Table 3 presents the seven selected BCTs mapped to key TDF domains.

**Table 3:** Final selection of BCTs to target each key domain and include as components of an intervention to improve medicines management for people with dementia (PwD) in primary care

Key TDF domain	Behaviour change techniques (BCTs) selected to target the TDF domain
Knowledge	Health consequences^a^
Skills	Modelling/demonstration of behaviour by others^b^
Memory, attention and decision processes	Self-monitoring^b^ Planning, implementation^b^ (equivalent to ‘Action planning’)
Behavioural regulation	Self-monitoring of behaviour^a^ Planning, implementation^b^ (equivalent to ‘Action planning’)
Social/professional role and identity	Social processes of encouragement, pressure, support^b^
Beliefs about capabilities	Self-monitoring^b^ Social processes of encouragement, pressure, support^b^
Beliefs about consequences	Salience of consequences^a^ Social and environmental consequences^a^ Self-monitoring^b^
Goals	Action planning (including implementation intentions)^a^ Social processes of encouragement, pressure, support^b^
Reinforcement	None selected^c^
Emotion	None selected^c^
Environmental context and resources	None selected^c^
Social influences	Modelling or demonstrating the behaviour^a^ Social process of encouragement, pressure, support^b^ Modelling/demonstration of behaviour by others^b^

^a^Identified from Cane et al. (2012) mapping tables [34]

^b^Identified from Michie et al. (2008) mapping tables [35]

^c^None of the BCTs mapped to these domains were considered to be feasible to target within the confines of the current project

No BCTs were selected for three of the key domains: ‘Reinforcement’, ‘Emotion’, and ‘Environmental context and resources’. Whilst a number of BCTs were identified against each of these domains, the BCTs were not considered feasible to target within the confines of the project, given the time and resources available and the primary care settings in which the intervention was to be implemented (Additional file 5).

### Draft intervention development

Two draft interventions operationalising selected BCTs were developed, targeting GPs (prescribing and conducting medication review) and community pharmacists (monitoring adherence and conducting medication review) respectively (Additional file 6). As carer involvement in medicines management was deemed to be critical by both HCP groups, both interventions were in the context of a consultation with a PwD and their carer. An online video was chosen to deliver the BCT ‘Modelling or demonstrating the behaviour’ in both interventions. This decision was informed by a recent project which had utilised a similar approach [22, 36] deemed acceptable by GPs in that study [39]. As both HCP groups in the current study had highlighted the time pressures they faced when managing medicines for PwD, it was envisaged that a video would not take up too much time and be readily accessible to HCPs working in busy clinical settings. It was anticipated that video content could be informed by findings from previous pharmacoepidemiological research [7]. The inclusion of a mentoring system or online discussion forum to deliver the BCT ‘Social processes of encouragement, pressure, support’ was informed by our interview findings, as some HCPs (particularly community pharmacists) had discussed their isolation from other colleagues. Such systems may allow HCPs to discuss difficult cases in a confidential manner, and receive guidance from peers.

### Task group work and selection of final intervention

Two task groups were conducted during December 2017 comprising GPs (*n* = 4; two of whom had previously participated in an interview) and community pharmacists (*n* = 5; all previous interview participants) respectively. The key strengths and limitations of the draft interventions, identified by participants during their discussions and application of the APEASE criteria, are shown in Table 4.

**Table 4:** Summary of strengths and limitations of draft interventions identified by task group participants

	GP-based intervention	Community pharmacy-based intervention
Strengths	• Likely to be an acceptable and practicable intervention. • One video preferred to multiple versions; preference for focus on medication review than prescribing. • Preference for resources to be made available online rather than paper-based; however online system must be easy to access and simple to navigate.	• Likely to be an affordable, practicable and acceptable intervention. • Presence of carer helpful to reduce patient anxiety/ reliance on patient report of information. • Mentoring system or online forum positively received. Links with local practice-based pharmacist would be useful and would help to strengthen and co-ordinate connections between GP and community pharmacist.
Limitations	• Due to heterogeneity among dementia patients in terms of staging/severity and medication issues, it will need to be clear to whom the intervention is aimed if it is to be effective (video may need to be tailored for different stages/severities). • Action planning document not considered to be acceptable. • Mentoring system not considered practical, as regular meetings already take place within practices and similar systems are already in place, particularly in large GP surgeries.	• Due to heterogeneity among dementia patients in terms of staging/severity and medication issues, it will need to be clear to whom the intervention is aimed if it is to be effective (video may need to be tailored for different stages/severities). • Time constraints if only one pharmacist on staff – pharmacists may not always be able to watch the video during working hours. Video must be concise.
Suggestions	• A ‘protocol’ should be developed to complement the video, which could include key information on contraindications and drug interactions, and which could be referred to when prescribing or conducting a medication review with a PwD. • Use of webinars or online discussion forums with multidisciplinary input suggested instead of mentoring systems.	• One video of no more than 15 min’ duration would be most practical. As it could reach a wider audience, it may also be cost-effective. • Further suggestions for ‘protocol’ content, e.g. common instances of potentially inappropriate prescribing, useful resources for healthcare professionals or for signposting patients/carers.

Following discussion within the research team, the community pharmacy-based intervention was selected for further feasibility testing [40]. The GP-based intervention was not considered for a number of reasons. Firstly, in the time since data collection had taken place, there had been a number of staff changes within the GP practices, making it difficult to re-engage with practices prior to the task groups. Secondly, the primary care organisational landscape in NI had changed significantly since data collection began. It was felt such issues would create additional difficulties in securing the participation of GP practices in a future feasibility study (our intention was to conduct feasibility work in sites which had been involved with the project from the outset).

Based on feedback provided during the task groups, the community pharmacy-based intervention was modified slightly to incorporate a complementary ‘protocol’ (termed a ‘quick reference guide’; QRG) document. Informal mentorship of community pharmacists by practice-based pharmacists was added, as alternatives (e.g. formal mentoring schemes, multidisciplinary webinars, online discussion forums) were beyond the scope of current project resources. Components of the final intervention and potential mechanisms of action [41] are outlined in Table 5.

**Table 5:** Summary of modified community pharmacy-based intervention selected for further feasibility testing

Description	Embedded BCT(s)	Mechanisms of action
A short online video demonstrating how a community pharmacist would conduct a medication review (incorporating adherence checking) with a PwD and their carer. The video would feature an authentic clinical case, incorporating relevant epidemiological data [7] and drawing upon clinical experience of research team. The positive outcomes of the consultation would be emphasised by including feedback from the pharmacist, PwD and their carer.	Modelling or demonstration of behaviour Health consequences Salience of consequences Social and environmental consequences	Skills, social influences, knowledge, beliefs about consequences
A complementary ‘quick reference guide’ (also made available online) to which pharmacists could refer during the medication review and adherence check. This guide would provide information on, e.g. common instances of potentially inappropriate prescribing, common drug interactions with drugs prescribed for dementia, guidance regarding antipsychotic drug use, tips on communicating with PwD, practice points on monitoring adherence in PwD, and useful sources of further information.	Modelling or demonstration of behaviour	Skills, social influences
After the pharmacist had watched the video and read the ‘quick reference guide’, they would identify suitable dementia patients from the pharmacy computer system and schedule an appointment for a PwD and their carer to attend the pharmacy for a face-to-face medication review and adherence check.	Action planning	Memory, attention and decision processes, behavioural regulation, goals
Following the review, the pharmacist would complete a clinical record form outlining any changes to the patient’s medication that they recommended. These would be shared with the patient’s GP and recorded on the pharmacy PMR so that the pharmacist could clearly see if their recommendations had been implemented by the GP.	Self-monitoring of behaviour	Memory, attention and decision processes, behavioural regulation, beliefs about capabilities, beliefs about consequences
Pharmacists would also be encouraged to liaise with the practice-based pharmacist for support and guidance during the process (e.g. to help resolve any issues arising from the medication review/adherence check).	Social processes of encouragement, pressure, support	Social/professional role and identity, beliefs about capabilities, goals, social influences

## Discussion

This study took a systematic approach to the development of an intervention to improve medicines management for PwD in primary care. In doing so, we have added to the body of work that has followed MRC guidance during the intervention development process and we have sought to address the lack of theory-based medicines management interventions described within the literature [22].

This study has provided a deeper understanding of issues of concern to primary HCPs about medicines management for PwD. This has helped to not only extend the evidence base in this area, but also ensured that our attention was focused on these issues during intervention development. To our knowledge, there are only a small number of published studies which aimed to elicit HCPs’ views about medicines management for PwD [42–44]. Our study has identified some similar barriers and facilitators to medicines management to those already identified within the literature, such as the importance of a multidisciplinary approach, the critical role of carers, the potential of medication reviews to improve medicines management, and pharmacists’ lack of access to clinical records [42–44]. However, these studies focused on the general concept of ‘medication management’ rather than the behaviours specific to each HCP group. In addition, the use of a structured theoretical framework in the current study helped us to identify barriers and facilitators that have not been mentioned previously, such as those relating to the clinical environment, access to resources, the processes by which HCPs’ attention is focused on medicines management, and their clinical decision-making in this area.

The very broad concept of medicines management, in hindsight, created additional complexity during the study. The definition of medicines management that was used [1] spans a number of components, and therein a number of different (and potentially target) ‘behaviours’. This resulted in us having to consider multiple behaviours during data collection and analysis. An alternative strategy would have been to identify and define a ‘problem’ and ‘target behaviour’ more specifically at the start of the study (e.g. focus on adherence in PwD). However, during study planning there was such a lack of literature in the area that we felt it was necessary to explore HCPs’ experiences and perspectives relating to the overarching concept of medicines management, in order to understand the problem through a wider lens. Producing narratives for each HCP group greatly helped us to reflect upon relevant behaviours, and to define and identify the ‘target behaviours’ [27, 45].

With the exception of ‘Intentions’ and ‘Optimism’, all of the theoretical domains (12 out of 14) were considered relevant to the target behaviours (i.e. prescribing and conducting medication review by GPs, and conducting medication review and monitoring adherence by community pharmacists). This illustrates the complex nature of the target behaviours, as well as the challenge faced by researchers in identifying and prioritising key domains to target when developing behaviour change interventions [46]. It was difficult to determine how the ‘Intentions’ and ‘Optimism’ domains influenced GPs’ and community pharmacists’ behaviour; these domains were also amongst the least frequently discussed by interview participants. Other studies exploring prescribing for older people have also found these domains to not be relevant [47, 48]. In selecting key domains, we noted that some of the barriers and facilitators reported by HCPs impacted on a number of different domains. Identifying a broadly similar group of domains for both HCP groups highlights the commonalities in the perceived mediators of behaviour change within each group. An overlap in the BCTs forming the components of the intervention involving GPs and/or pharmacists was unsurprising, given that the same key domains were selected and this has been encountered by other researchers [22, 23]. Having identified the challenges of busy clinical environments in the primary care setting through the qualitative interviews (e.g. time and workload pressures), we selected BCTs that were likely to be most potent and not require repeated administration to elicit the required changes in the target group’s behaviour. However, access to greater resources may permit inclusion of BCTs that we were unable to in the current study, for example by incorporating an incentive or reward (monetary or otherwise) for HCPs delivering the intervention (targeting the ‘Reinforcement’ domain), or through enhanced access for community pharmacists to patient health records via IT infrastructure (targeting the ‘Environmental context and resources’ domain).

Having been through a rigorous but lengthy analytical and intervention development process, the task groups gave the research team the opportunity to explore how proposed intervention components could be implemented in clinical practice [37, 38]. Implementation of complex interventions in primary care is known to be challenging, and the literature highlights the importance of paying attention to context during implementation [49, 50]. The task groups helped us to consider many elements in relation to this such as external context, organisation and professional issues, as well as the intervention itself [49]. Task group participants’ feedback was invaluable, with many helpful and pragmatic suggestions regarding the draft interventions. For example, the action-planning component that was suggested by the research team was not regarded to be useful by GPs, and the concept of a ‘protocol’ (which became the QRG) was initially suggested by GPs and supported by community pharmacists. It is hoped that this additional ‘stage’ of the intervention development process will help to ensure that the components of the final intervention can be feasibly and pragmatically incorporated into routine community pharmacy practice. This will be tested in a future feasibility study in a small number of community pharmacies.

The project was conducted at a time of great change within primary care in NI, with the creation of new practice-based pharmacist roles in GP surgeries [51]. While the activities undertaken by these pharmacists are reported to be wide-ranging and variable, many of their tasks are focused on outcomes related to medicines optimisation [52]. Given that some of the GP participants referred to the potential for practice-based pharmacists to contribute to optimising medicines management for PwD, this will be an area for future research.

### Strengths and limitations

This study has produced rich, descriptive data about participants’ involvement in medicines management for PwD from the perspectives of two primary HCP groups. In transparently reporting the steps taken and experiences encountered during this work, we have added to the body of evidence on operationalising the TDF and BCT mapping. The systematic and robust approach taken to analysis and intervention development ensures that the final intervention will be both evidence- and theory-based. The use of a theoretical framework to inform the development of behaviour change interventions is recommended [22], and there is a distinct lack of theory-based medicines management interventions for PwD in primary care [17]. As the final intervention proceeds through feasibility and pilot testing it may undergo further refinement, helping to improve the chances of successful implementation and benefits to the target population. The contribution of HCP stakeholders throughout the intervention development process has already been recognised; their involvement will ensure that intervention components address issues of importance to the end user and are relevant to, and applicable in, daily practice. Inputs from each member of the multidisciplinary research team have been valuable particularly during interpretation of the data from clinical and psychological viewpoints. As with all research studies, there are a number of limitations. Qualitative findings must be interpreted in light of the study context and setting; findings may not be applicable to other settings and geographical areas. In addition, participants may reflect those with a strong interest in, and awareness of, medicines management and their participation was incentivised. The data presented in this study represent the perceptions of HCPs interviewed, and therefore are subject to any reporting biases that are likely to be pertinent to HCPs in this context and at this time. The possibility of interviewer bias must be considered when interpreting the findings, however steps were taken to minimise this through adopting a reflexive interviewing style, conducting regular debriefing sessions during data collection, and the analytical approach (independent coding by two researchers, with regular meetings to discuss and agree upon coding).

## Conclusions

This study has highlighted the complexities of medicines management for PwD from the perspectives of primary HCPs. Our findings have provided a wider evidence-base for complex intervention development in this area. A community pharmacy-based intervention has been developed targeting medicines management for PwD in primary care using a systematic, theory-informed approach. This study used task group methodology during the intervention development process; it is hoped that this will improve future uptake and implementation of the intervention. Further work will focus on feasibility testing and possible refinement of this intervention, before a larger pilot trial may proceed.

## Supplementary information

**Additional file 1.** Description of the 14 theoretical domains from the TDF. Descriptions of the theoretical domains that were used to inform topic guide development and code interview data.

**Additional file 2.** GP interview topic guide. TDF-based topic guide used during GP interviews that was used to explore theoretical domains as barriers and facilitators to medicines management for PwD in primary care.

**Additional file 3.** Community pharmacist interview topic guide. TDF-based topic guide used during community pharmacist that was used to explore theoretical domains as barriers and facilitators to medicines management for PwD in primary care.

**Additional file 4.** Healthcare professional narratives. Descriptions of each of the identified target behaviours for both healthcare professional groups.

**Additional file 5.** BCT mapping. Mapping of behaviour change techniques (BCTs) to key domains for inclusion in an intervention to improve medicines management for PwD in primary care

**Additional file 6. **Draft intervention outlines. Description of draft interventions to improve medicines management for PwD in primary care*. (DOCX 24 kb)*

## Data Availability

The data that supports the findings of this study are available on reasonable request from the corresponding author. The data are not publicly available due to them containing information that could compromise research participant privacy/consent.

## Abbreviations

BCT: Behaviour change technique
BPSD: Behavioural and psychological symptoms of dementia
GP: General practitioner
HCP: Healthcare professional
HSC: Health and Social Care
MRC: Medical Research Council
NICRN: Northern Ireland Clinical Research Network
PwD: Persons with dementia
QRG: Quick reference guide
TDF: Theoretical Domains Framework
