# Supplementary material for: Improving medicines management for people with dementia in primary care: a qualitative study of healthcare professionals to develop a theory-informed intervention
Source: BMC Health Serv Res. 2020 Feb 14;20:120. doi: 10.1186/s12913-020-4971-7 (PMC7023803; doi:10.1186/s12913-020-4971-7)
Supplement: Supplementary file 1 — Additional file 1. Description of the 14 theoretical domains from the TDF. Descriptions of the theoretical domains that were used to inform topic guide development and code interview data. [file 12913_2020_4971_MOESM1_ESM.docx]

| Domain label | Definition | Theoretical constructs represented within each domain |
| --- | --- | --- |
| Knowledge | *An awareness of the existence of something* | Knowledge (including knowledge of condition/scientific rationale); procedural knowledge; knowledge of task environment |
| Skills | *An ability or proficiency acquired through practice* | Skills; skills development; competence; ability; interpersonal skills; practice; skill assessment |
| Memory, attention and decision processes | *The ability to retain information, focus selectively on aspects of the environment and choose between two or more alternatives* | Memory; attention; attention control; decision-making; cognitive overload/tiredness |
| Behavioural regulation | *Anything aimed at managing or changing objectively observed or measured actions* | Self-monitoring; breaking habit; action planning |
| Social/professional role and identity | *A coherent set of behaviours and displayed personal qualities of an individual in a social or work setting* | Professional identity; professional role; social identity; identity; professional boundaries; professional confidence; group identity; leadership; organisational commitment |
| Beliefs about capabilities | *Acceptance of the truth, reality, or validity about an ability, talent, or facility that a person can put to constructive use* | Self-confidence; perceived competence; self-efficacy; perceived behavioural control; beliefs; self-esteem; empowerment; professional confidence |
| Optimism | *The confidence that things will happen for the best or that desired goals will be attained* | Optimism; pessimism; unrealistic optimism; identity |
| Beliefs about consequences | *Acceptance of the truth, reality, or validity about outcomes of a behaviour in a given situation* | Beliefs; outcome expectancies; characteristics of outcome expectancies; anticipated regret; consequents |
| Intentions | *A conscious decision to perform a behaviour or a resolve to act in a certain way* | Stability of intentions; stages of change model; Transtheoretical model and stages of change |
| Goals | *Mental representations of outcomes or end states that an individual wants to achieve* | Goals (distal/proximal); goal priority; goal/target setting; goals (autonomous/controlled); action planning; implementation intention |
| Reinforcement | *Increasing the probability of a response by arranging a dependent relationship, or contingency, between the response and a given stimulus* | Rewards (proximal/distal, valued/not valued, probable/improbable); incentives; punishment; consequents; reinforcement; contingencies; sanctions |
| Emotion | *A complex reaction pattern, involving experiential, behavioural, and physiological elements, by which the individual attempts to deal with a personally significant matter or event* | Fear; anxiety; affect; stress; depression; positive/negative affect; burn-out |
| Environmental context and resources | *Any circumstances of a person’s situation or environment that discourages or encourages the development of skills and abilities, independence, social competence, and adaptive behaviour* | Environmental stressors; resources/material resources; organisational culture/climate; salient events/critical incidents; person x environment interaction; barriers and facilitators |
| Social influences | *Those interpersonal processes that can cause individuals to change their thoughts, feelings, or behaviours* | Social pressure; social norms; group conformity; social comparisons; group norms; social support; power; intergroup conflict; alienation; group identity; modelling |
