# Supplementary material for: Improving medicines management for people with dementia in primary care: a qualitative study of healthcare professionals to develop a theory-informed intervention
Source: BMC Health Serv Res. 2020 Feb 14;20:120. doi: 10.1186/s12913-020-4971-7 (PMC7023803; doi:10.1186/s12913-020-4971-7)
Supplement: Supplementary file 2 — Additional file 2. GP interview topic guide. TDF-based topic guide used during GP interviews that was used to explore theoretical domains as barriers and facilitators to medicines management for PwD in primary care. [file 12913_2020_4971_MOESM2_ESM.docx]

**GP INTERVIEW TOPIC GUIDE**

**Development of an intervention to improve medicines management for people with dementia in primary care in Northern Ireland**

**Introduction**

“*My name is <researcher name>, and I am a researcher from the School of Pharmacy, Queen’s University Belfast. Thank you very much for making the time to speak with me today.*

*In this research project we are interested in finding out what medicines people with dementia are taking, and how they manage those medicines. In the first part of this project we analysed prescriptions received by patients in Northern Ireland during the year 2013, using prescribing data from the Business Services Organisation. We used a set of prescribing criteria to explore the appropriateness of prescribing for these patients. Now we are speaking to patients with dementia and their carers to explore their views and experiences of medicines management in more detail. We are also interviewing GPs and community pharmacists as they are responsible for prescribing and dispensing medicines for this patient population, and may have different views and experiences to patients and their carers. We plan to use all of the information we gather during this study to see if we can develop an intervention to improve medicines management for people with dementia, which will be tested for feasibility in the final part of the project.*

*Have you had a chance to read through the information sheet that was sent out to you? Are there any questions that you would like to ask me before we start?”*

**Explaining what will happen in the interview and afterwards**

*“The aim of this interview is to explore your views of medicines management in people with dementia, your approach to prescribing for this patient population, and your perceptions of the barriers and facilitators to successful medicines management for people with dementia in primary care. I’d like to focus specifically on people with dementia living within the community as opposed to those in nursing or residential care home settings. The interview should last approximately <*estimated duration*> minutes.*

*I will be recording the interview on a digital recorder, to ensure that we have an accurate and detailed record of what you say. The recording will be saved on a password-protected computer and only those immediately involved in the research study will listen to them. The recording will be typed up word-for-word and any names, locations, or anything else that could identify you will be removed so that the information is anonymous. After we have conducted interviews with all of the other participants we will analyse the information within the research team.*

*You are free to stop the interview and/or recording at any point. If there are any questions that you would prefer not to answer, just let me know and we can move on to the next question.*

*Before we start I need to get written consent from you that you understand what the study involves; anything you say will be kept completely confidential; you will not be identified in any way; we can stop the interview at any time; and also that you are happy for the interview to be recorded. If you wouldn’t mind, can you read through the consent form and initial each box to indicate that you understand and agree with each statement? There are two copies: you will keep one of them and I will keep the other for our records.*

*Have you any immediate questions about the study before we start the interview?”*

[Turn the digital recorder on]

**Demographic information**

- Can you tell me how long you have been practising as a GP?
- Have you completed any additional training (either formal or self-directed) in dementia?
- Approximately, what proportion of the patients in this practice have a diagnosis of dementia?
- On a typical working day in your practice, approximately:
  - How many dementia patients would you encounter (e.g. through face-to-face or over the telephone consultations)?
  - How many carers of dementia patients would you encounter?
- Approximately what proportion of your overall prescribing is for patients with dementia?
- What would be the average number of items regularly prescribed per dementia patient?

**Definitions**

*“There is no widely-accepted definition of medicines management, although the term is often used. For the purpose of this project, we are adopting a definition of medicines management used by the Audit Commission (2001) which states that:*

[Hand interview participant a card with definition printed on it]

*‘Medicines management encompasses the entire way that medicines are selected, procured, delivered, prescribed, administered, and reviewed to optimise the contribution that they make to producing informed and desired outcomes of patient care’*

*In short, the essential components of medicines management are prescribing, dispensing, administration, adherence, and medication review. The cornerstone of medicines management is ensuring that patients gain maximum benefit from their medicines, whilst also minimising the risk of harm.”*

Social/professional role and identity

- Thinking about medicines management for patients with dementia, what would you consider your contribution/responsibilities to be as a GP in ensuring that patients with dementia and their carers are able to manage medicines appropriately and effectively?*
  - **Prompt:** Is there anything that you would consider to be beyond your contribution/ responsibility as a GP (in ensuring that patients with dementia and their carers can manage their medicines appropriately and effectively)?
  - **Prompt:** Who do you think is responsible for these aspects beyond your contribution/responsibility?

***Note:** The participant’s answer to this question will determine how subsequent questions are asked/worded (see below for sections shaded in grey)

**Behavioural elicitation**

*“It would be helpful if you can think of a situation where you have prescribed medication and been responsible for the subsequent management of a patient with dementia. You may also have dealt with the patient’s carer. For the rest of the questions I ask you, it might be useful to keep this example in mind. If you can’t think of a specific situation, don’t worry, just think about medicines management for patients with dementia in general terms using the definition I’ve given you.”*

Knowledge

- What knowledge do you think you need as a GP when <prescribing medicines for/ assessing adherence of/ conducting medication reviews in> patients with dementia?
  - **Prompt:** Clinical knowledge?
    - Specific knowledge sources/resources
    - Is there anything specifically relating to prescribing/adherence/medication review in patients with dementia?
  - **Prompt:** Knowledge of the patient’s clinical picture
  - **Prompt:** Knowledge of guidelines (specific to dementia)?
    - What guidelines?
    - What do such guidelines recommend?
  - **Prompt:** Personal knowledge/experience of dementia?
    - What effect does this have on your clinical practice?

Skills

- What skills do you have as a GP to assist you when <prescribing medicines for/ assessing adherence of/ conducting medication reviews in> patients with dementia and dealing with any issues that may arise with <prescribing/ adherence/ medication review> in such patients?
  - **Prompt:** What skills do you have that would help you to engage with patients or their carers?
  - **Prompt:** What skills do you have that would help you to engage with other healthcare professionals?
- Is there any specific training which you feel would be helpful to you in order to improve <prescribing/ adherence/ medication review> for patients with dementia in the future?

Beliefs about capabilities

- In what situations do you feel confident about <prescribing medicines for/ assessing adherence of/ conducting medication reviews in> patients with dementia?
- In what situations do you **not** feel confident about <prescribing medicines for/ assessing adherence of/ conducting medication reviews in> patients with dementia?
  - **Prompt:** What would help you to overcome these problems or difficulties?

Optimism

- How optimistic are you that appropriate <prescribing/ adherence/ medication review> can be achieved for patients with dementia?
- What would make you feel less optimistic that appropriate <prescribing/ adherence/ medication review> can be achieved for patients with dementia?

Beliefs about consequences

- What do you think are the benefits of appropriate <prescribing/ adherence/ medication review> for patients with dementia?
  - **Prompt:** For patients; their carers; yourself; NHS; short and long-term consequences
- What do you think are the risks associated with inappropriate <prescribing/ adherence/ medication review> for patients with dementia?

Reinforcement

- What would encourage you to ensure <prescribing/ adherence/ medication review> is appropriate for patients with dementia?
  - **Prompt:** Are there any rewards or incentives for you or the practice, e.g. QOF, personal rewards, professional recognition?
- What would discourage you from improving <prescribing/ adherence/ medication review> for patients with dementia?

Intentions

- How do you plan (intend) to address issues with <prescribing/ adherence/ medication review> for patients with dementia?
- What would prevent you from addressing issues with <prescribing/ adherence/ medication review> for patients with dementia?

Goals

- To what extent is improving <prescribing/adherence/medication review/educating or counselling> for patients with dementia a priority for you?
  - If low/high priority, why?
- In what circumstances would you think it was less important to make any changes to <prescribing/ adherence/ medication review> for patients with dementia?

Memory, attention and decision processes

- How would you usually remember to address issues with <prescribing/ adherence/ medication review> for patients with dementia?
  - **Prompt:** For example, if there was an issue with patient adherence/ prescribing antipsychotic medications/ patient increasingly struggling with medicines but living alone?
- Are there any circumstances in which you might just forget or find it difficult to resolve these issues?
  - **Prompt:** How would you describe the complexity of decision-making in <prescribing/ adherence/ medication review> for patients with dementia?

Environmental context and resources

- What resources or support might help you to intervene when you encounter issues with <prescribing/ adherence/ medication review> in patients with dementia?
- What factors might prevent you from intervening when you encounter issues with <prescribing/ adherence/ medication review> in patients with dementia?
  - **Prompt:** Work environment and culture within practice; material resources available; critical incidents/events within the practice

Social influences

- Who would influence your decisions about dealing with issues with <prescribing/ adherence/ medication review> for patients with dementia?
  - **Prompt:** Patients; carers or relatives; community pharmacist; hospital consultant; colleagues within the practice/surgery; other healthcare professionals
  - **Prompt:** Can you tell me more about how this happens and what their influence is?

Emotion

- How does <prescribing/ supporting adherence/ performing medication review> for patients with dementia make you feel?
- How would your own work stress or emotional engagement with a patient and their carer influence your decisions to attempt to resolve issues with <prescribing/ adherence/ medication review> for patients with dementia?

Behavioural regulation

- Having decided the best course of action to resolve <prescribing/ adherence/ medication review> issues for a patient with dementia, are there any ways in which you can monitor whether or not it has been done?
  - **Prompt:** Following clinical guidelines or workplace protocols
- What strategies would you use to overcome these circumstances?

**Intervention components**

*“The research team is interested in developing an intervention to support and improve medicines management for people with dementia and their carers. From reviewing the literature, we have found that interventions can be complex and often involve a number of different components. This makes it difficult to identify which components are the most important in terms of improving patient outcomes and achieving adequate medicines management.”*

- What would you consider to be important components of an intervention to improve medicines management for people with dementia and their carers in primary care?
- **Prompt:** Who should be involved in delivering these types of interventions in practice (e.g. carers, community pharmacists, GPs, practice nurses, voluntary sector)?
- **Prompt:** What would each person/healthcare professional have to do?
- **Prompt:** What are your thoughts on patient involvement in interventions – should patients be actively involved in the decisions about the medicines they are prescribed?
- What would be the facilitators to putting the type of intervention that you have described into practice?
- What would be the barriers to putting the type of intervention that you have described into practice?
- What would help the implementation of the intervention?
- What do you think should be measured as an outcome in an intervention study to support medicines management for people with dementia, i.e. how would you, personally, be persuaded that the intervention had improved medicines management? What are the important outcomes?

**Closing the interview**

*“That brings us to the end of the interview.*

*Is there anything else about medicines management in people with dementia that you feel has not been covered?*

*Do you have any additional comments you would like to make as to the content of the interview or how it went?*

*Thank you very much for making the time to speak with me today.”*

[Turn the digital recorder off]
