# Supplementary material for: Improving medicines management for people with dementia in primary care: a qualitative study of healthcare professionals to develop a theory-informed intervention
Source: BMC Health Serv Res. 2020 Feb 14;20:120. doi: 10.1186/s12913-020-4971-7 (PMC7023803; doi:10.1186/s12913-020-4971-7)
Supplement: Supplementary file 4 — Additional file 4. Healthcare professional narratives. Descriptions of each of the identified target behaviours for both healthcare professional groups. [file 12913_2020_4971_MOESM4_ESM.docx]

| **TARGET BEHAVIOUR** | PRESCRIBING |
| --- | --- |
| *Who* **needs to perform the behaviour?** | General Practitioners |
| *What* **do they need to do differently to achieve the desired change?** | Ensure they are prescribing appropriately for people with dementia. Special consideration must be given to potentially inappropriate medications/combinations identified during Phase 1 work |
| *When* **do they need to do it?** | At any time when prescribing a new or repeat medication for a dementia patient, which could be:  - during a face-to-face consultation  - following a telephone call  - when signing off repeat prescriptions |
| *Where* **do they need to do it?** | In the GP surgery |
| *How often* **do they need to do it?** | Any time they are prescribing |
| *With whom* **do they need to do it?** | Alone or in conjunction with the patient and/or patient’s carer (depending upon situation) |

| **TARGET BEHAVIOUR** | CONDUCTING MEDICATION REVIEW |
| --- | --- |
| *Who* **needs to perform the behaviour?** | General practitioners  Community pharmacists |
| *What* **do they need to do differently to achieve the desired change?** | Conduct regular and comprehensive medication review* for dementia patients, following a structured and systematic process  *Medication review will also include an assessment of appropriateness of prescribing and adherence |
| *When* **do they need to do it?** | During medication review appointment |
| *Where* **do they need to do it?** | In the GP surgery, pharmacy, or at the patient’s home |
| *How often* **do they need to do it?** | Initial review following diagnosis  Annual review thereafter? |
| *With whom* **do they need to do it?** | Alone* or with the patient and/or their carer  *There may be some elements of medication review that could take place prior to GP/pharmacist discussion with patient and carer |

| **TARGET BEHAVIOUR** | MONITORING ADHERENCE |
| --- | --- |
| *Who* **needs to perform the behaviour?** | Community pharmacists |
| *What* **do they need to do differently to achieve the desired change?** | Ensure that they are regularly checking patient adherence to medication (PMR check, asking questions of patient and/or carers, checking previous compliance aid, making home visit) when they are dispensing medication for people with dementia |
| *When* **do they need to do it?** | Routinely during dispensing process |
| *Where* **do they need to do it?** | In the pharmacy and/or in the patient’s home (in order to make an accurate assessment of adherence) |
| *How often* **do they need to do it?** | Monthly*  *Home visit not required monthly, but could be arranged e.g. twice a year |
| *With whom* **do they need to do it?** | Alone, and with input from the patient and/or their carer |
