# Supplementary material for: Improving medicines management for people with dementia in primary care: a qualitative study of healthcare professionals to develop a theory-informed intervention
Source: BMC Health Serv Res. 2020 Feb 14;20:120. doi: 10.1186/s12913-020-4971-7 (PMC7023803; doi:10.1186/s12913-020-4971-7)
Supplement: Supplementary file 5 — Additional file 5. BCT mapping. Mapping of behaviour change techniques (BCTs) to key domains for inclusion in an intervention to improve medicines management for PwD in primary care [file 12913_2020_4971_MOESM5_ESM.docx]

| **Theoretical domain** | **BCTs identified from Cane *et al*. (2015) [34]** | **BCTs identified from Michie *et al*. (2008) [35]** | **Selected BCTs as proposed intervention components (including reasons to justify exclusion of other BCTs** |
| --- | --- | --- | --- |
| *Knowledge* | 1. Health consequences 2. Biofeedback 3. Antecedents 4. Feedback on behaviour | 1. Information regarding behaviour, outcome | Health consequences (BCT 1): HCPs will be provided with information detailing and emphasising the health benefits of performing the behaviour. There will need to be a focus on what will happen if the behaviour is performed and not performed.  Reasons for not selecting other BCTs:  BCT 2: not applicable as feedback about the body using an external monitoring device is unlikely to have an impact on the target behaviours.  BCT 3: intervention would likely need to be tailored to individual HCPs to account for variation in emotions, cognitions, social and environmental situations that would predict performance of the behaviour.  BCTs 4, 5: likely to require repeated administration and/or extended time periods to effect required changes in target behaviours. |
| *Skills* | 1. Graded tasks 2. Behavioural rehearsal/practice 3. Habit reversal 4. Body changes 5. Habit formation | 1. Goal/target specified: behaviour or outcome 2. Monitoring 3. Self-monitoring 4. Rewards; incentives (inc. self-evaluation) 5. Graded task starting with easy task 6. Increasing skills: problem-solving, decision-making, goal-setting 7. Rehearsal of relevant skills 8. Modelling/demonstration of behaviour by others 9. Homework 10. Perform behaviour in different settings | Modelling/demonstration of behaviour by others (BCT 13): HCPs would be provided with a demonstration of how to perform the behaviour during a typical encounter/consultation with a PwD and their carer.  Reasons for not selecting other BCTs:  BCTs 1, 2, 3, 5, 7, 8, 10, 12, 14: likely to require repeated administration and/or extended time periods to effect required changes in target behaviours.  BCT 4: Not applicable as a direct change in HCPs; body structure/functioning is unlikely to have an impact on the target behaviours.  BCT 6: not possible to establish an acceptable goal/target in terms of the number of PwD that HCPs would perform target behaviours on because ideally the target behaviours should be performed on all PwD.  BCT 9: not within scope of project to offer rewards/incentives.  BCT 11: intervention would likely need to be tailored to individual HCPs to account for baseline variation in skill levels.  BCT 15: not applicable as the intervention will target HCPs in their normal place of work. |
| *Memory, attention and decision processes* | No BCTs linked to this domain | 1. Self-monitoring 2. Planning, implementation 3. Prompts, triggers, cues | Self-monitoring (BCT 1): HCPs would be asked to record whether they have performed the target behaviour(s) and review this at designated intervals to ensure that medication issues are actioned.  Planning, implementation (BCT 2; equivalent to ‘Action planning’): HCPs would be encouraged to plan in detail their performance of the target behaviour(s) in advance of performing them.  Reasons for not selecting other BCTs:  BCT 3: not within scope of project to introduce environmental or social stimuli with the purpose of prompting or cueing the target behaviours. |
| *Behavioural regulation* | 1. Self-monitoring of behaviour | 1. Goal/target specified: behaviour or outcome 2. Contract 3. Planning, implementation 4. Prompts, triggers, cues 5. Use of imagery | Self-monitoring of behaviour (BCT 1): see under ‘Memory, attention and decision processes’ domain.  Planning, implementation (BCT 4): see under ‘Memory, attention and decision processes’ domain.  Reasons for not selecting other BCTs:  BCT 2: not possible to establish an acceptable goal/target in terms of the number of PwD that HCPs would perform the target behaviours on, because ideally the target behaviours should be performed on all PwD.  BCT 3: not within the scope of the project to impose additional contractual obligations on HCPs.  BCT 5: see under ‘Memory, attention and decision processes’ domain.  BCT 6: used in the context of implementing other BCTs through the use of planned images (visual, motor, sensory); not applicable in the context of this research project. |
| *Social/professional role and identity* | No BCTs linked to this domain | 1. Social processes of encouragement, pressure, support | Social processes of encouragement, pressure, support (BCT 1): HCPs would be encouraged to seek support/mentorship from other colleagues and/or primary healthcare professionals which would encourage and support them in engaging with PwD and their carers to improve medicines management. For example, community pharmacists would be encouraged to seek support from the local general practice-based pharmacist. |
| *Beliefs about capabilities* | 1. Verbal persuasion to boost self-efficacy 2. Focus on past successes | 1. Self-monitoring 2. Graded task, starting with easy task 3. Increasing skills: problem-solving, decision-making, goal-setting 4. Coping skills 5. Rehearsal of relevant skills 6. Social processes of encouragement, pressure, support 7. Feedback 8. Self-talk 9. Motivational interviewing | Self-monitoring (BCT 3): see under ‘Memory, attention and decision processes’ domain.  Social processes of encouragement, pressure, support (BCT 8): see under ‘Social/professional role and identity’ domain.  Reasons for not selecting other BCTs:  BCTs 1, 10: intervention would likely need to be tailored to individual HCPs to account for baseline variation in self-efficacy levels.  BCT 2: not suitable due to potential variation in experience amongst HCPs (i.e. if HCPs do not have previous experience of performing the target behaviours then this BCT will not apply to them).  BCTs 4, 9: likely to require repeated administration and/or extended time periods to effect required changes in target behaviours.  BCTs 5, 6, 7: intervention would likely need to be tailored to individual HCPs to account for baseline variation in skills levels.  BCT 11: not within scope of project to offer motivational interviewing to individual HCPs. |
| *Beliefs about consequences* | 1. Emotional consequences 2. Salience of consequences 3. Covert sensitization 4. Anticipated regret 5. Social and environmental consequences 6. Comparative imagining of future outcomes 7. Vicarious reinforcement 8. Threat 9. Pros and cons 10. Covert conditioning | 1. Self-monitoring 2. Persuasive communication 3. Information regarding behaviour, outcome 4. Feedback | Salience of consequences (BCT 2) and Social and environmental consequences (BCT 5): HCPs will be provided with information from HCPs, PwD and carers emphasising the social and environmental benefits of performing the behaviours. There will need to be a memorable focus on what will happen if the behaviour is performed and not performed.  Self-monitoring (BCT 11): see under ‘Memory, attention and decision processes’ domain.  Reasons for not selecting other BCTs:  BCT 1: emotional consequences of performing the target behaviours have not been established.  BCTs 3, 4: not applicable as intervention is focused on wanted behaviours as opposed to unwanted behaviours.  BCT 6: intervention would likely need to be tailored to individual HCPs as the imagining and comparing of future outcomes of changed versus unchanged behaviour is likely to vary between individuals.  BCTs 7, 10, 14: likely to require repeated administration and/or extended time periods to effect required changes in target behaviours.  BCT 8: not within scope of project to implement future punishment or removal of reward as a consequence of HCPs performing an unwanted behaviour.  BCT 9: intervention would likely need to be tailored to individual HCPs because if advised to identify and compare pros and cons of performing the target behaviours, assessments are likely to vary between individuals.  BCT 12: difficult to have a credible source present evidence-based arguments in favour of or against the target behaviours as few interventions to date have examined clinically relevant outcomes.  BCT 13: intervention would likely need to be tailored to individual HCPs to account for baseline variation in skills levels when advising on how to perform the target behaviours. |
| *Goals* | 1. Goal setting (outcome) 2. Goal setting (behaviour) 3. Review of outcome goal(s) 4. Review behaviour goals 5. Action planning (including implementation intentions) | 1. Goal/target specified: behaviour or outcome 2. Contract 3. Rewards, incentives (inc. self-evaluation) 4. Graded task, starting with easy task 5. Increasing skills: problem-solving, decision-making, goal-setting 6. Social processes of encouragement, pressure, support 7. Persuasive communication 8. Information regarding behaviour, outcome 9. Motivational interviewing | Action planning (BCT 5): see under ‘Memory, attention and decision processes’ domain; equivalent to planning, implementation BCT.  Social processes of encouragement, pressure, support (BCT 11): see under ‘Social/professional role and identity’ domain.  Reasons for not selecting other BCTs:  BCTs 1, 2, 6: not possible to establish an acceptable goal/target in terms of the target behaviours to be achieved or number of PwD that HCPs would perform target behaviours on because ideally the target behaviours should be performed on all PwD.  BCTs 3, 4: not possible to review behaviour or outcome goals if acceptable goals not set/established (as per BCTs 1, 2 above).  BCT 7: See under ‘Behavioural regulation’ domain.  BCTs 8, 9, 10: See under ‘Skills’ domain.  BCTs 12, 13: See under ‘Beliefs about consequences’ domain  BCT 14: See under ‘Beliefs about capabilities’ domain |
| *Reinforcement* | 1. Threat 2. Self-reward 3. Differential reinforcement 4. Incentive 5. Thinning 6. Negative reinforcement 7. Shaping 8. Counter conditioning 9. Discrimination training 10. Material reward 11. Social reward 12. Non-specific reward 13. Response cost 14. Anticipation of future rewards or removal of punishment | Domain not included in matrix | Reasons for not selecting BCTs:  BCT 1: see under ‘Beliefs about consequences’ domain.  BCT 2: difficult to have a HCP reward self with material or other valued object(s) if effort and/or progress has been made in performing the target behaviours.  BCTs 3, 5, 6, 7, 8, 9, 13: not within scope of project to implement future punishment, removal of reward or reinforcement for performing or not performing target behaviours.  BCTs 4, 10, 11, 12, 14: see under ‘Skills’ domain (equivalent to ‘Rewards, incentives BCT). |
| *Emotion* | 1. Reduce negative emotions 2. Emotional consequences 3. Self-assessment of affective consequences 4. Social support (emotional) | 1. Stress management 2. Coping skills | BCT 4: encapsulated by ‘Social processes of encouragement, pressure, support’ BCT under ‘Social/professional role and identity’ domain.  Reasons for not selecting BCTs:  BCTs 1, 3, 5, 6: intervention would likely need to be tailored to individual HCPs to account for variation in levels of emotions, stress and coping skills associated with performance of target behaviour.  BCT 2: see under ‘Beliefs about consequences’ domain. |
| *Environmental context and resources* | 1. Restructuring the physical environment 2. Discriminative (learned) cue 3. Prompts/cues 4. Restructuring the social environment 5. Avoidance/changing exposure to cues for the behaviour | 1. Environmental changes (e.g. objects to facilitate behaviour) | Reasons for not selecting BCTs:  BCTs 1, 6: not within the scope of the project to restructure HCPs’ physical work environment.  BCT 2: not within the scope of project to offer reward (e.g. monetary fee) for performing target behaviours.  BCT 3: see under ‘Memory, attention and decision processes’ domain.  BCT 4: not within the scope of project to restructure HCPs’ social environment.  BCT 5: not applicable as intervention is seeking to promote performance of target behaviours as opposed to avoiding/reducing exposure to cues for the target behaviours. |
| *Social influences* | 1. Social comparison 2. Social support or encouragement (general) 3. Information about others’ approval 4. Social support (emotional) 5. Social support (practical) 6. Vicarious reinforcement 7. Restructuring the social environment 8. Modelling or demonstrating the behaviour 9. Identification of self as role model 10. Social reward | 1. Social process of encouragement, pressure, support 2. Modelling/demonstration of behaviour by others | Social support or encouragement (BCT 2)/ Social process of encouragement, pressure, support (BCT 11): see under ‘Social/professional role and identity’ domain.  Modelling or demonstrating the behaviour/ Modelling/demonstration of behaviour by others (BCT 12): see under ‘Skills’ domain.  Reasons for not selecting BCTs:  BCT 1: difficult to draw meaningful comparisons between HCPs’ performance of target behaviours.  BCT 3: difficult to establish PwD’s views on HCPs performing the target behaviours due to clinical heterogeneity amongst PwD in terms of comorbidities and medications used.  BCTs 4, 5: encapsulated by BCT 2.  BCT 6: see under ‘Beliefs about consequences’ domain.  BCT 7: see under ‘Environmental context and resources’ domain.  BCT 9: likely to require repeated administration and/or extended time periods to effect required changes in HCPs’ behaviours.  BCT 10: see under ‘Skills’ domain (equivalent to ‘Rewards, incentives BCT). |
