# Supplementary material for: Improving medicines management for people with dementia in primary care: a qualitative study of healthcare professionals to develop a theory-informed intervention
Source: BMC Health Serv Res. 2020 Feb 14;20:120. doi: 10.1186/s12913-020-4971-7 (PMC7023803; doi:10.1186/s12913-020-4971-7)
Supplement: Supplementary file 6 — Additional file 6. Draft intervention outlines. Description of draft interventions to improve medicines management for PwD in primary care. (DOCX 24 kb) [file 12913_2020_4971_MOESM6_ESM.docx]

| **GENERAL PRACTICE-BASED INTERVENTION** |
| --- |
| **Target group:** GPs  **Target behaviours:** Prescribing and conducting medication review  **Intervention description:** The intervention would be delivered through a short online video (or series of videos) demonstrating how GPs can prescribe appropriately during a typical consultation with a PwD and their carer (‘Modelling or demonstrating of behaviour’). Elements of a medication review conducted by the GP would also be incorporated in the video (or as a separate video). Each video would include feedback from the GP, the PwD and their carer emphasising the positive outcomes of the consultation (‘Health consequences’, ‘Salience of consequences’, ‘Social and environmental consequences’).  As complementary intervention components, GPs would be provided with an action planning template to help them make an explicit plan of when, where, and how they would carry out each behaviour, and with whom (‘Action planning’). GPs would be encouraged to regularly review this to ensure their behaviour is monitored and recorded (‘Self-monitoring of behaviour’). A one-to-one mentoring system would be implemented to facilitate action planning and to provide GPs with the opportunity to discuss challenging clinical cases with GP colleagues (‘Social processes of encouragement, pressure, support).  **Mechanisms of action:** Beliefs about capabilities, knowledge, subjective norms, intention, social influences, attitude towards the behaviour, behavioural regulation and skills |
| **COMMUNITY PHARMACY-BASED INTERVENTION** |
| **Target group:** Community pharmacists  **Target behaviours:** Monitoring adherence and conducting medication review  **Intervention description:** The intervention would be delivered through a short online video (or series of videos) similar to the GP-based intervention described above. The video would operationalise a number of BCTs (‘Modelling or demonstrating of behaviour’, ‘Health consequences’, ‘Salience of consequences’, ‘Social and environmental consequences’) by demonstrating how a pharmacist could monitor adherence and conduct a medication review during a scheduled consultation with a PwD and their carer. The video would include feedback from the pharmacist, PwD and their carer emphasising the positive outcomes of this consultation.  Patients would be identified by the pharmacist from a search of the pharmacy patient medication record (PMR) and pharmacists would plan to approach PwD and their carers when they would next present at the pharmacy. If agreeable, pharmacists would schedule an appointment to conduct the adherence check and medication review (‘Action planning’). Upon completion of this, pharmacists would ensure that any changes they had recommended to patients’ medications were communicated to GPs, and recorded on the PMR (‘Self-monitoring of behaviour’).  In order to provide the opportunity for confidential discussion with other community pharmacists, a one-to-one mentoring system or online discussion forum would be incorporated as a complementary intervention component (‘Social processes of encouragement, pressure, support’).  **Mechanisms of action:** Beliefs about capabilities, knowledge, subjective norms, intention, social influences, attitude towards the behaviour, behavioural regulation and skills |
